# The Attenuating Effect of Beta-Carotene on Streptozotocin Induced Diabetic Vascular Dementia Symptoms in Rats

**DOI:** 10.3390/molecules27134293

**Published:** 2022-07-04

**Authors:** Khian Giap Lim, Rajavel Varatharajan, Arunachalam Muthuraman

**Affiliations:** Faculty of Pharmacy, Asian Institute of Medicine, Science and Technology University, Bedong 08100, Kedah, Malaysia; khiangiap96@hotmail.com (K.G.L.); varadharajeen@gmail.com (R.V.)

**Keywords:** acetylcholinesterase, lipid peroxidation, Morris water maze, reduced glutathione, thiobarbituric acid

## Abstract

This study investigated the ameliorative effects of beta-carotene (BC) on diabetes-associated vascular dementia and its action against biomolecule oxidation. The diabetic vascular dementia (VaD) was induced by administration of nicotinamide (NA; 50 mg/kg; *i.p.*) and streptozotocin (STZ; 50 mg/kg; *i.p.*). The test compound, BC (50 and 100 mg/kg; *p.o.*), and the reference compound, donepezil (DP) (1 mg/kg; *p.o.*), were administered for 15 consecutive days. Changes in learning and memory were assessed by escape latency time (ELT) and times spent in target quadrant (TSTQ) in the Morris water maze (MWM) test. The changes in neurotransmitter, i.e., acetylcholinesterase (AChE) and oxidative stress markers, i.e., thiobarbituric acid reactive substance (TBARS) and reduced glutathione (GSH), were estimated in hippocampal tissue of the rat brain. The administration of STZ caused significant deterioration of cognitive function (decreased ELT and raised the TSTQ) as compared to the normal group. Treatment with BC and DP diminished the increased AChE activity, TBARS level and decreased GSH level caused by STZ. Thus, BC ameliorates the diabetic vascular complications in VaD due to its potential anticholinergic, antioxidative and free radical scavenging actions.

## 1. Introduction

Lifestyle-related diseases are becoming more common in this modern era. One of the most common noncommunicable diseases is dementia. There are many subtypes of dementia, with vascular dementia (VaD) being the second most common one [1]. In VaD, vascular injuries happen in the important brain regions that are involved in memory, cognition and behavior. VaD impairs the quality of life of those affected and incurs enormous healthcare costs, apart from increasing the morbidity and causing disabilities [2]. Therefore, the treatment and prevention of VaD should be the cornerstone in clinical research. Nevertheless, it is very challenging to standardize the diagnostic criteria of VaD, as several factors are involved in the pathogenesis of dementia and these factors may interact with each other as well. Some of these factors include the origin, location, volume, timing, number of lesions and systemic factors, such as hypertension and hyperglycemia [3]. To make matters worse, most of the dementia cases have mixed pathology, comprising features of Alzheimer’s disease (AD) (amyloid plaques and neurofibrillary tangles), as well as ischemic lesions [4]. Generally, patients with VaD usually experience deterioration in attention span and executive function, such as reduced capacity to solve problems, impaired tasks execution, inability to plan, disorientation and poor judgement [1,5]. Further, VaD patients may also have slowed thinking, poor reasoning or even depression and anxiety [6].

The complexity of the pathogenesis of VaD and lack of ideal animal models to study VaD halts the discovery of effective medications to treat this syndrome. VaD is heterogenous in nature, where the lesions formed involve different brain areas that are critical for cognitive function. These lesions disrupt the basal ganglia cortical, cortico-cortical and ascending pathways [7]. To date, there is no medication approved for the treatment of VaD. Beta carotene (BC) is a red-orange color pigment commonly found in natural food sources, such as carrots, tomatoes, red pepper, animal liver and tuna [8]. Biologically, it acts as a precursor for vitamin A with the help of BC 15,15′-monooxygenase [9]. It has potent protective action for the neurovascular system, with potent free radical scavenging action [10]. Furthermore, it reduces the expression of inflammatory factors, i.e., nuclear factor kappa-B (NF-kβ), interleukins and inducible nitric oxide synthases (iNOS) levels [11]. There are limited studies on the role of supplementation of BC in memory impairments via regulation of the neurovascular system. Therefore, we intend to investigate the effect of BC on VaD.

## 2. Results

### 2.1. Effect of Beta Carotene on Escape Latency Time and Time Spent in Target Quadrant in the Morris Water Maze Test

**Figure 1:**
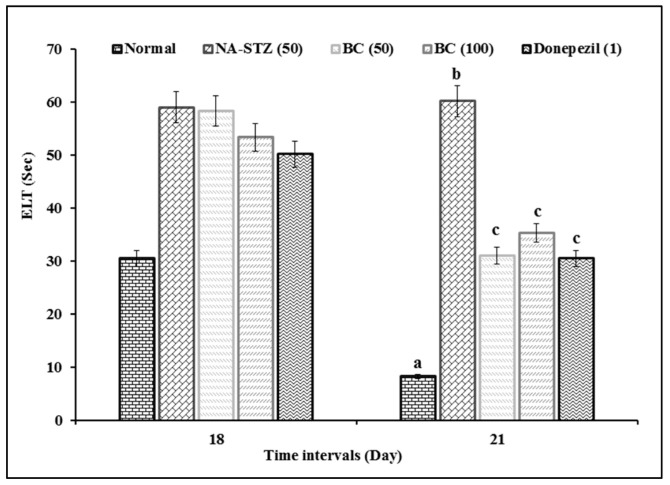
Effect of BC on ELT in MWM. Digits in parenthesis indicated dose in mg/kg. Data were expressed as mean ± SD; *n* = 12 rats. ^a^ *p* < 0.05 vs. ELT of normal group on day 18; ^b^ *p* < 0.05 vs. ELT of normal group on day 21; and ^c^ *p* < 0.05 vs. ELT of VaD group on day 21. Abbreviations: BC, beta-carotene; ELT, escape latency time; NA, nicotinamide; and STZ, streptozotocin.

**Figure 2:**
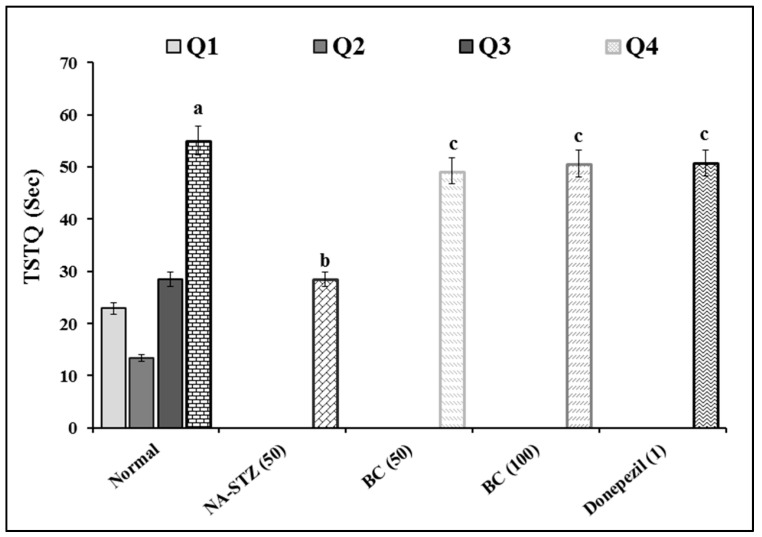
Effect of BC on TSTQ in MWM. Digits in parenthesis indicated dose in mg/kg. Data were expressed as mean ± SD; *n* = 12 rats. ^a^ *p* < 0.05 vs. the time spent in Q1 of normal group; ^b^ *p* < 0.05 vs. TSTQ (Q4) of normal group; ^c^ *p* < 0.05 vs. TSTQ (Q4) of VaD group. Abbreviations: BC, beta-carotene; NA, nicotinamide; Q, quadrant; STZ, streptozotocin; TSTQ, time spent in target quadrant.

The administration of BC showed a significant (*p* < 0.05) ameliorative effect against the diabetic VaD condition in the MWM test. There is a significant difference in ELT between groups [F (4,25) = 38.777; *p* < 0.001] and between days [F (3,75) = 125.04; *p* < 0.001]. Further, the interaction between groups and days is significant [F (12,75) = 7.354; *p* < 0.001]. On day 18, there is no difference in ELT across groups, except normal group. Rats in normal group showed significant reduction in ELT from day 18 to day 21. This showed that normal rats have normal learning ability. However, VaD control group had longer ELT compared to all other groups from day 18 to day 21. The TSTQ (Q4) of normal rats is longer on day 22 when compared to other quadrants (Q1, Q2, and Q3) [F (3,15) = 29.608; *p* < 0.001]. Hence, normal rats have significant memory retention ability (increased in TSTQ) on day 22. The TSTQ between groups showed significant difference [F (4,25) = 11.167; *p* < 0.001], where the TSTQ of VaD control rats on day 22 was longer than that of other groups. This indicates that the VaD control group had potential impairment of memory retention. The treatment with BC (50 and 100 mg/kg) and DP (1 mg/kg) showed a significant (*p* < 0.001) ameliorative effect against the NA-STZ-induced increased ELT and decreased TSTQ. Therefore, both BC and DP attenuated the NA-STZ-induced impairment of learning and memory retention (Figure 1 and Figure 2). There is no statistically significant difference in ELT and TSTQ between groups treated with BC and DP.

### 2.2. Effect of Beta Carotene on Tissue Biomarkers

In VaD control group, the AChE activity and the level of thiobarbituric acid reactive substance (TBARS) were increased, while the level of reduced glutathione (GSH) was decreased in the hippocampus when compared to the normal control group, as shown in Table 1. The one-way ANOVA of AChE activity showed statistically significant difference between groups: F (4,25) = 30.277; *p* < 0.001. Similarly, the levels of GSH [F (4,25) = 24.73; *p* < 0.001] and TBARS [F (4,25) = 15.882; *p* < 0.001] were significantly different between groups. Treatment with BC (50 and 100 mg/kg) and DP (1 mg/kg) hampered the alteration of above tissue biomarkers in NA-STZ-induced diabetic VaD. Hence, BC and DP potentially attenuate the VaD-associated changes in tissue biomarkers via regulation of cellular oxidative stress and enzymatic activity.

## 3. Discussion

Our study showed that the VaD control group had longer ELT and shorter TSTQ in the MWM test, increased AChE activity and TBARS level, as well as reduced GSH level compared to the normal group. This indicates that the administration of STZ resulted in impairment of spatial memory and an increase in oxidative stress. The administration of BC and DP improved the performance in behavioral test and, hence, the spatial memory of animals in MWM tests. This finding was associated with attenuation of VaD-induced changes in AChE activity and oxidative stress marker levels. VaD is heterogenous in nature and involves complex mechanisms that are involved in the pathophysiology. Generally, VaD is a result of decreased blood flow to certain parts of the brain [12], which causes ischemic lesions to occur in neuronal networks supplying the brain. Multiple facets (mitochondrial dysfunction [13], endothelial dysfunction [14], blood brain barrier disruption [15], white matter damage [16], and neuroinflammation [17]) are involved throughout the development and progression of VaD. The cholinergic system is crucial for cognition, including execution, memory and emotion control [18]. Deficiency of this system is associated with significant decline in executive function [19]. Cholinergic dysfunction most commonly occurs in the basal forebrain cholinergic nuclei and reduces cholinergic connections to the cortex [12]. Cholinergic dysfunction may also be due to a reduction in affinity of receptor towards ligand. It was reported that cerebral blood flow (CBF) and cholinergic signaling affect each other reciprocally [20]. The widespread WM and vascular lesions result in disruption of cholinergic connections, ultimately leading to dysfunction of the cholinergic system. This contributes to the decrease in CBF and, subsequently, brain hypoperfusion [21]. Impairment of the cholinergic system was evident in studies of VaD [18,22,23], where there was deafferentation of frontal and limbic cortical structures, as well as disturbance of basal ganglia, thalamus, white matter and sub frontal areas [24]. In VaD, there are loss of cholinergic neurons, reduced choline acetyltransferase (ChAT) activity, decrement in m3 and m5 muscarinic acetylcholine (ACh) receptor expression, lowered ACh level and worsened memory and learning [25].

Apart from the cholinergic system, the regulation of oxidative stress is also crucial to the development and progression of VaD [26]. In our study, we used STZ for the induction of diabetic VaD. STZ was known to alter the cerebral oxidative stress as well as energy metabolism through impairment of adenosine triphosphate (ATP) and acetyl CoA synthesis [27]. STZ enters pancreatic beta-cells via GLUT 2 glucose transporter [28]. The pancreas is very susceptible to oxidative damage by STZ because of the low level of antioxidant enzymes [29]. Further, STZ also reduces the expression of nuclear factor-erythroid factor 2-related factor 2 (Nrf2), which is responsible for regulating antioxidative response. This happens owing to the increased reactive oxygen species (ROS) and the ratio of oxidized glutathione (GSSG) to GSH [30]. In a study investigating the molecular mechanisms of STZ damage on pancreatic beta-cells, it was found that STZ inhibits the mitochondrial respiratory enzymes. Parallelly, there is an increase in the generation of ROS and reactive nitrogen species (RNS), causing lipid peroxidation. On top of that, caspase-3 and caspase-9 are activated, which leads to DNA fragmentation and apoptosis [31]. Diabetes is one of the risk factors of VaD. It hinders the hippocampal long-term potentiation, and hence impairs learning ability as well as memory. Diabetes also decreases the level of nitric oxide (NO), which leads to impaired vasodilation and reduced CBF [32]. In diabetic condition, there is increased production of ROS, which attack the membranous polyunsaturated fatty acids. Consequently, there is an increased level of malondialdehyde, which is reflected by increased TBARS level [33]. GSH is one of the most potent endogenous antioxidants. Its production requires nicotinamide adenine dinucleotide phosphate (NADPH). However, diabetes leads to an increased level of reduced nicotinamide adenine dinucleotide (NADH). This, in turn, depletes the NADPH and, therefore, decreases GSH level [34].

Although the frontal cortex was not investigated in this study, it plays an important role in spatial working memory. In a study using a photothrombosis-induced frontal cortex stroke mice model, the authors reported that neuronal cell degeneration, reactive astrogliosis and infiltration of immune cells occurred in the frontal cortex, which ultimately leads to behavioral impairment [35]. In another study using frozen post-mortem human brains of VaD, AD and mixed dementia, it was found that the extent of hypoperfusion in frontal cortex was most significant in VaD brains. The myelin-associated glycoprotein:proteolipid protein-1 (MAG:PLP-1) ratio was inversely associated with level of endothelin-1 and proportional to the level of vascular endothelial growth factor (VEGF) in frontal cortex [36]. Our finding is in line with a study conducted in 2019, where BC improved the spatial memory of rats with obstructive sleep apnea syndrome, as evident in shorter ELT in the MWM test when compared to the disease group. This is due to a decrease in the expression of caspase-3 and phosphorylated tau (pτ) protein [37]. The administration of BC at a dose of 2.05 mg/kg inhibited the increase in AChE activity in STZ-induced Alzheimer’s disease in mice. The authors also performed in silico docking studies and proved that BC has high binding affinity towards AChE [38]. Another enzyme that is related to AChE is butyrylcholinesterase (BuChE). BuChE is claimed to be involved in the cell proliferation and growth of nervous system. In cases of AD, the level of BuChE correlates with the expression of neuritic plaques and neurofibrillary tangles [39]. BuChE is also involved in neuroinflammation, as the cholinergic anti-inflammatory pathway regulates the expression of proinflammatory cytokines, such as tumor necrosis factor alpha, interleukin-1, interleukin-6 and Prostaglandin E2 [40]. Hence, it is worth incorporating BuChE in future studies.

In a study using an animal model of two-kidney one-clip (2K1C) renovascular hypertension-induced vascular dementia, there is an increase in TBARS level and decrease in the GSH level in the disease control group [41]. In another study using a mice model of STZ-induced AD, the administration of all-trans retinoic acid resulted in improved memory, reduction in STZ-induced increase in AChE activity, TBARS and reduction in GSH [42]. BC is a reddish orange color pigment and it belongs to the carotenoid family. It consists of eight isoprene units and is rich in conjugated double bond [43]. BC has potent antioxidative activity due to the presence of double bonds. BC can chelate ROS and RNS through energy transfer during the formation and cleavage of bonds [44]. It was also reported that BC can reduce membrane lipid peroxidation by suppressing the expression of inflammatory cytokines and nuclear factor-kappa β (NF-κβ) [45]. Interestingly, BC possesses neuroprotective activity by modulating the Nrf2/Kelch-like ECH-associated protein 1 (KEAP1) pathway [46], where the expression of Nrf2 increases, while the expression of KEAP1, repressor of Nrf2, decreases [47]. Therefore, BC could ameliorate VaD through regulation of oxidative stress.

## 4. Materials and Methods

### 4.1. Animals

Disease-free healthy male Sprague Dawley rats (220 ± 20 g) were obtained from Lab-rat Breeders Farm PVT Ltd., Selangor, Malaysia, and housed in Central Animal House of AIMST University, Malaysia. The conditions of Central Animal house were maintained as follows: temperature (22 ± 1 °C), relative humidity (60%) and 12-h light/dark cycle. The animals had access to food and water ad libitum and were kept for 7 days for adaptation before starting the experiment. All the animal handling, dosing, behavioral assessments as well as sacrifice were conducted during daytime.

### 4.2. Induction of Diabetic Vascular Dementia

The animals were first administered with nicotinamide (NA; 50 mg/kg) through the intraperitoneal (*i.p.*) route, followed by streptozotocin (STZ; 50 mg/kg; *i.p.*), with a 15 min interval in between for the induction of diabetic VaD [48,49]. Tail vein blood samples were taken on the 3rd and 7th day for measurement of non-fasting blood glucose level. Animals with a blood glucose level of more than 200 mg/dL were considered diabetic and were used in this study [50].

### 4.3. Experimental Protocol

The diabetic animals were distributed into 5 groups of 12 animals each, with different interventions.

Group 1: Normal, healthy rats (normal control).

Group 2: NA (50 mg/kg; *i.p.*), followed by (15 min later) STZ (50 mg/kg; *i.p.*) were administered for the induction of VaD (disease control group).

Group 3 and 4: BC (Nacalai Tesque Inc.) (50 and 100 mg/kg; for 15 consecutive days) was administered orally (*p.o.*) for the treatment of VaD (induced animal) for the respective group (treatment groups).

Group 5: DP (1 mg/kg; *p.o.*; for 15 consecutive days) was administered per oral (*p.o.*) for the treatment of VaD (induced animal) (reference group).

**Figure 3:**
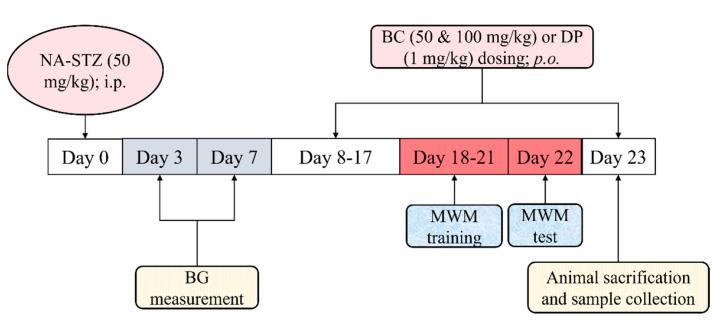
The experimental protocol. VaD was induced by NA and STZ injection on day 0. The blood glucose levels were measured on day 3 and day 7. BC and DP were administered from day 8 to day 23. The MWM training (learning phase) was conducted from day 18 to day 21. The MWM test (retrieval phase) was conducted on day 22. All the animals were sacrificed on day 23. Abbreviations: NA, nicotinamide; STZ, streptozotocin; BC, beta-carotene. DP, donepezil; BG, blood glucose; EPM, elevated plus maze; *i.p.*, intraperitoneal, *p.o.*, per oral; mg/kg, milligram per kilogram.

The details of the experimental protocol are illustrated in Figure 3. The dose of BC was chosen based on the fact that rodents have a good capacity to convert BC into vitamin A. Hence, they can be fed a large amount of BC. In a study that developed a novel BC formulation, BC at a dose of 50 mg/kg was administered to rats for 7 days [51]. To the best of our knowledge, there is no research article published on the investigation of BC in the rat model of VaD.

### 4.4. Collection of Biological Samples

Briefly, the animals were anesthetized with diethyl ether and sacrificed by cervical dislocation. The head was isolated and the skull was cut open to collect the brain samples. Then, the hippocampus was isolated from the whole brain [52]. Each hippocampus was homogenized (10% *w*/*v*) with phosphate buffer saline (pH 7.4) and subjected to centrifugation at 3500 rpm (1720 g) for 15 min [53]. The supernatant obtained was used for biochemical assessment.

### 4.5. Assessment of Spatial Learning and Memory by Morris Water Maze

The MWM test was conducted as described in the method of Morris [54]. Briefly, a circular water pool (150 cm in diameter; 45 cm in height) was used. The water pool was divided into 4 quadrants with distinct visual cues on the inner wall of each quadrant. A platform (10 × 10 cm square and 28 cm in height) was placed in one of the quadrants and water was filled until the water level was 2 cm above the platform. During the training session, each animal was placed in each quadrant to assess the escape latency time (ELT): the time needed to find the hidden platform. Each animal was allowed to explore the pool for 120 s. Once the animals found the platform, they were allowed to stay on the platform for 15 s. Otherwise, they were guided to the platform after 120 s. During the MWM test day, each animal was placed in the middle of the pool and allowed to swim for 120 s. The time spent in the target quadrant (TSTQ) by each animal was noted and it served as the index of retrieval (memory retention) [55].

### 4.6. Estimation of Acetylcholinesterase (AChE) Activity

The hippocampal AChE activity was estimated as described in the method of Ellman et al. [56]. Concisely, 0.5 mL of brain supernatant was added with 2.5 mL of phosphate buffer saline (pH 7.4). Thereafter, 0.1 mL of 5,5-dithio-bis-(2-nitrobenzoic acid) (DTNB) solution was added. The changes in absorbance (optical density; OD) were measured spectrophotometrically (Shimadzu UV-1800 UV/Visible Scanning Spectrophotometer, Kyoto, Japan) at 412 nm wavelength. Next, 20 μL of acetylthiocholine iodide was added. The changes in absorbance were measured instantly and at an interval of every 2 min until the absorbance value became constant. The acetylcholinesterase activity was calculated using the following formula:

$$\mathrm{Acetylcholinesterase}\;\mathrm{activity}\;=\frac{∆A}{13,600}\times \frac{1}{\left(\frac{\mathrm{VBs}}{\mathrm{TVts}}\right)P}$$

where ∆*A*, changes in absorbance/min; *P*, protein content (mg/mL); VBs, volume of brain supernatant (VBs = 500 µL); TVts, total volume of test samples (TVts = 3120 µL); and 13,600 refers to the molar extinction coefficient of DTNB (M^−1^ cm^−1^). The AChE activity results were expressed as μM of acetylthiocholine hydrolyzed/mg of protein/minute.

### 4.7. Estimation of Reduced Glutathione (GSH)

The GSH content was estimated using the method of Beutler et al. [57]. Briefly, the aliquots (0.5 mL) were mixed with 2 mL of 0.3 M disodium hydrogen phosphate. Thereafter, 0.25 mL of 0.001 M freshly prepared DTNB (5, 5′-dithiobis (2-nitrobenzoic acid) dissolved in 1% *w*/*v* sodium citrate) was added. The changes in absorbance were noted spectrophotometrically (Shimadzu UV-1800 UV/Visible Scanning Spectrophotometer, Kyoto, Japan) at 412 nm. A standard curve was plotted using 10–100 µM of reduced form of glutathione. The results of GSH were expressed as micromoles of GSH per mg of protein.

### 4.8. Estimation of Thiobarbituric Acid Reactive Substances (TBARS)

The TBARS content was estimated using the method of Buege and Aust [58]. Briefly, 0.2 mL of brain supernatant was mixed with 2 mL of thiobarbituric acid (TBA), trichloroacetic acid (TCA), and hydrochloric acid (HCL) reagent mixture. The mixture was heated for 10 min in a boiling water bath to develop pink colour chromogen. Thereafter, tubes were cooled with tap water and centrifuged at 5500 rpm (3252 g) for 15 min. The changes in absorbance were noted spectrophotometrically (Shimadzu UV-1800 UV/Visible Scanning Spectrophotometer, Kyoto, Japan) at 532 nm wavelength. A standard curve was plotted using 0–100 µM of tetramethoxypropane. The results of TBARS were expressed as micromoles of TBARS per mg of protein.

### 4.9. Statistical Analysis

The data collected for both EPM test and AChE activity estimation were expressed as mean ± standard deviation (SD) and analyzed by two-way and one-way analysis of variance (ANOVA) test correspondingly. The post hoc analysis was performed using Tukey’s honestly significant difference (HSD) test. The statistical analysis was conducted using Statistical Package for the Social Sciences (SPSS) software (version 25). The alpha value was set at 0.05.

## 5. Conclusions

Our study showed that BC improves the performance of rats in MWM and attenuates the elevated AChE activity, TBARS level and reduced GSH level. These signify that BC, as a natural compound that is widely available, is helpful in ameliorating VaD. As ACh is a crucial neurotransmitter that regulates bodily functions, it is worth exploring and investigating the potential of BC in other neurodegenerative diseases in which the cholinergic system is affected. This study can also be extrapolated to a clinical stage once more fruitful results are reported.

## Figures and Tables

**Table 1:** Effect of BC on hippocampal biomarkers.

Groups	ACHE (μmol of Acetylthiocholine Iodide/min/mg of Protein)	GSH (μmol/L/mg of Protein)	TBARS (μM/mg of Protein)
Normal	159.73 ± 29.05	35.68 ± 3.47	1.05 ± 0.16
NA-STZ	311.21 ± 26.58 ^a^	23.53 ± 2.54 ^a^	1.67 ± 0.12 ^a^
BC (50)	227.11 ± 18.82 ^a,b^	31.41 ± 2.45 ^b^	1.18 ± 0.16 ^b^
BC (100)	234.56 ± 20.97 ^a,b^	33.51 ± 2.36 ^b^	1.05 ± 0.22 ^b^
DP (1)	231.29 ± 22.60 ^a,b^	35.88 ± 1.10 ^b^	1.01 ± 0.17 ^b^

Digits in parenthesis indicated dose in mg/kg. Data were expressed as mean ± SD; *n* = 12 rats. ^a^ *p* < 0.05 vs. normal group; ^b^ *p* < 0.05 vs. VaD group. Abbreviations: AChE, acetylcholinesterase; BC, beta-carotene; DP, donepezil; GSH, reduced glutathione; NA, nicotinamide; STZ, streptozotocin; TBARS, thiobarbituric acid reactive substances.

## Data Availability

The data presented in this study are available on request from the corresponding author.

## Sample Availability

Samples of the compounds are available from the authors.
